# ESGO/ESTRO quality indicators for radiation therapy of cervical cancer

**DOI:** 10.1136/ijgc-2022-004180

**Published:** 2023-05-29

**Authors:** Cyrus Chargari, Kari Tanderup, François Planchamp, Luis Chiva, Pauline Humphrey, Alina Sturdza, Li T Tan, Elzbieta van der Steen-Banasik, Ignacio Zapardiel, Remi A Nout, Christina Fotopoulou

**Affiliations:** 1 Institut Gustave-Roussy, Villejuif, France; 2 Aarhus University Hospital, Aarhus, Denmark; 3 Clinical Research Unit, Institut Bergonie, Bordeaux, France; 4 Obstetrics and Gynecology, Clinica Universidad de Navarra, Madrid, Spain; 5 University Hospitals Bristol and Weston NHS Foundation Trust, Bristol, UK; 6 Department of Radiation Oncology, Comprehensive Cancer Center, Christian Doppler Laboratory for Medical Radiation Research for Radiation Oncology, Medical University of Vienna, Wien, Austria; 7 Addenbrooke's Hospital, Cambridge, UK; 8 Radiotherapie Groep/Arnhem, Arnhem, The Netherlands; 9 Gynecologic Oncology, La Paz University Hospital, Madrid, Spain; 10 Radiotherapy, Erasmus MC Cancer Centre, Rotterdam, Netherlands; 11 Gynaecologic Oncology, Imperial College London Faculty of Medicine, London, UK

**Keywords:** cervical cancer

## Abstract

**Background:**

The European Society of Gynaecological Oncology (ESGO) has previously defined and established a list of quality indicators for the surgical treatment of cervical cancer. As a continuation of this effort to improve overall quality of care for cervical cancer patients across all aspects, ESGO and the European SocieTy for Radiotherapy and Oncology (ESTRO) initiated the development of quality indicators for radiation therapy of cervical cancer.

**Objective:**

To develop a list of quality indicators for radiation therapy of cervical cancer that can be used to audit and improve clinical practice by giving to practitioners and administrators a quantitative basis to improve care and organizational processes, notably for recognition of the increased complexity of modern external radiotherapy and brachytherapy techniques.

**Methods:**

Quality indicators were based on scientific evidence and/or expert consensus. The development process included a systematic literature search for identification of potential quality indicators and documentation of scientific evidence, consensus meetings of a group of international experts, an internal validation process, and external review by a large international panel of clinicians (n=99).

**Results:**

Using a structured format, each quality indicator has a description specifying what the indicator is measuring. Measurability specifications are detailed to define how the quality indicators will be measured in practice. Targets were also defined for specifying the level which each unit or center should be aiming to achieve. Nineteen structural, process, and outcome indicators were defined. Quality indicators 1–6 are general requirements related to pretreatment workup, time to treatment, upfront radiation therapy, and overall management, including active participation in clinical research and the decision making process within a structured multidisciplinary team. Quality indicators 7–17 are related to treatment indicators. Quality indicators 18 and 19 are related to patient outcomes.

**Discussion:**

This set of quality indicators is a major instrument to standardize the quality of radiation therapy in cervical cancer. A scoring system combining surgical and radiotherapeutic quality indicators will be developed within an envisaged future ESGO accreditation process for the overall management of cervical cancer, in an effort to support institutional and governmental quality assurance programs.

## Introduction

Cervical cancer is the fourth most common cancer worldwide. The average world age standardized incidence is 13 per 100 000 per year, with wide geographical variations. Approximately 570 000 cervical cancer cases occurred in 2018 in the world, with 311 000 deaths.^1^ The World Health Organization recently launched a cervical cancer elimination initiative, aiming to reduce its incidence to below 4 per 100 000 by the end of the century worldwide.^2^ This plan mainly relies on three strategies:

1. Vaccination against human papillomaviruses, which are responsible for 90% of all cervical cancers
2. Screening and treatment of detected cervical pre-invasive and invasive lesions;
3. Offering the best possible curative care to women diagnosed with invasive cancer.

Until such plans are successfully implemented, cervical cancer remains a significant healthcare issue across Europe and beyond, with wide national treatment variations. In East Europe, it is the most frequent cause of cancer death in women aged <44 years.^3^

Treatment indications for cervical cancer are available in joint guidelines published by the European Society of Gynaecological Oncology (ESGO), the European SocieTy for Radiotherapy and Oncology (ESTRO), and the European Society of Pathology (ESP).^4–6^ An update of these guidelines is currently in progress, with expected release by the end of 2022. These guidelines will provide an overview of the most recent evidence and recommendations for diagnosis, surgical treatment, and radiotherapy/systemic treatment in cervical cancer. Quality of treatment improvement and established quality indicators are crucial for the provision of optimal care, with a demonstrated benefit for both treatment related morbidity and oncologic safety. ESGO has previously defined and established a list of quality indicators for the surgical treatment of cervical cancer.^7^ As a continuation of this effort to improve overall quality of care for cervical cancer patients across all aspects, ESGO and ESTRO collaborated to extend the quality indicators and include aspects of radiation therapy management to develop a quality indicator program for accreditation of centers for cervical and overall cancer management.

These quality indicators aim to provide clinicians and healthcare authorities with a quantitative background of optimal standard of care and an evidence based framework for improving quality of care across Europe at institutional and national levels. The ultimate aim is to improve oncologic outcomes by minimizing treatment related morbidity and complications profiles, and to homogenize treatment care across Europe and beyond. These quality indicators are intended to give practitioners and administrators a quantitative basis to improve care and organizational processes notably for recognition of the increased complexity of modern external radiotherapy and brachytherapy techniques. They also facilitate the documentation of quality of care, comparison of performance structures, and establishment of organizational priorities as a basis for accreditation in European countries. The key characteristics of an ideal indicator are clear definition, clinical relevance, measurability, and feasibility in clinical practice. The quality indicators and proposed targets are based on the standards of practice determined from available scientific evidence and/or expert consensus.

The intention is incentive, not punitive. Certified centers can make the award known to doctors, patients, patient advocacy groups, and lay persons. Moreover, the targets defined by the international development group is not to be used to penalize or litigate doctors or institutions. These quality indicators will be updated in the future, based on new evidence, as appropriate. Even though our aim is to present the highest standard of evidence based care in an optimal treatment setting in qualified cervical cancer centers, ESGO, ESTRO, and the international development group acknowledge that there will be broad variability in practices across the various centers worldwide, with significant differences in infrastructure, access to medical, radiotherapeutic, and surgical advances, and technology. Moreover, the variation in training, medicolegal, financial, and cultural aspects may affect the implementation and applicability of any quality indicators in each country and healthcare system.

## Methods

**Figure 1:**
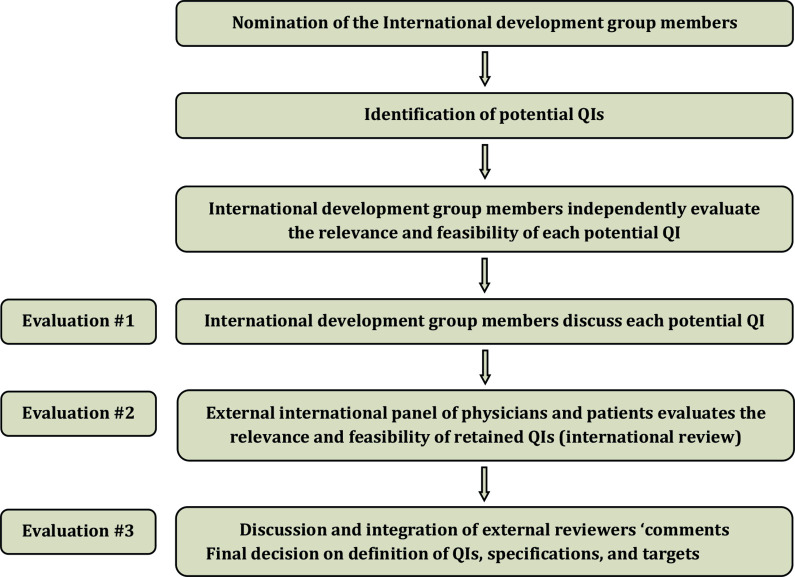
Development process and the three step evaluation process. QIs, quality indicators.

Quality indicators for radiation therapy of cervical cancer were developed using a three step evaluation process, including an unbiased and independent systematic literature search performed by an experienced methodologist, an ad hoc international development group chaired by Professor Cyrus Chargari (ESTRO, Gustave Roussy Cancer Campus, Villejuif, France) and Professor Christina Fotopoulou (ESGO, Imperial College London, UK), an internal validation process, and an external review by a large panel of expert clinicians of the ESTRO and ESGO network (Figure 1).

ESGO/ESTRO nominated five radiation oncologists, one physicist, one therapeutic radiographer, and three gynecologic oncologists from among ESGO and ESTRO members, whose expertise had been previously confirmed by identifying articles, oral presentations, administrative responsibilities, and other works of any type on leadership in improving the quality of care for patients with cervical cancer. All potential quality indicators, including external beam radiotherapy and brachytherapy, were identified from the ESGO–ESTRO–ESP guidelines.^4–6^ In addition, a systematic literature search was conducted in Medline without any restriction of the search period, using the following indexing terms: quality indicators, quality assurance, cervical cancer, cervix uteri, uterine neoplasms, methodology, consensus statements, radiation therapy, radiation oncology, intensity modulated radiotherapy, image guided radiotherapy, image guided brachytherapy, interstitial brachytherapy, concurrent chemotherapy, overall treatment time, and evidence based medicine. References were selected if those described indicators developed by other agencies or synthesized research evidence describing practice that contributed to improved patient outcomes (guidelines or consensus statements). Previous initiatives publishing quality indicators for radiotherapy quality of care indicators in cervical cancer were also identified.^8–11^

Potential quality indicators were formatted as a questionnaire and sent to the international development group. Experts were asked to evaluate each indicator according to relevance and feasibility in clinical practice. They were also free to propose any additional potential quality indicators they deemed relevant. Acceptance, rejection, or the need for further consideration of each indicator was discussed. Quality indicators were retained if a large consensus among experts was obtained. ESGO–ESTRO established a large panel of practicing clinicians who provide care to patients with cervical cancer. These international reviewers are independent of the international development group and are from different European and non-European countries to ensure global perspective. The retained quality indicators were formatted as a questionnaire and sent to the external reviewers for quantitative evaluation of each indicator according to relevance, feasibility in clinical practice, and quality of care improvement. Open comments were encouraged (qualitative evaluation). Evaluations of the quality indicators were returned by 99 independent physicians (Online Supplemental Appendix 1). Responses were pooled and sent to the international development group members and comments were reviewed to finalize the quality indicators' development process. Definitions of quality indicators, specifications, and targets were validated. Although the strengths of the process include an international development group, international expert consensus to support the quality indicators, an international external review process, a structured format to present the quality indicators, and management of potential conflicts of interests, the quality indicators result from a consensus of experts, with inherent bias in this type of method.

10.1136/ijgc-2022-004180.supp1 Supplementary data

## Results

Each retained quality indicator is categorized as a structural indicator, process indicator, or outcome indicator, and has a description which specifies what the quality indicator is measuring.^12^ The measurability specifications are then detailed. The latter highlight the way in which the quality indicator will be measured in practice to allow audits. The time frame for assessment of criteria is the last calendar year (unless otherwise indicated). Further to measurement of the indicator, a target is indicated. This specifies the level which each unit or center should be aiming to achieve. When appropriate, two targets were defined: an optimal target, expressing the best possible option for patients, and a minimal target, expressing the minimal requirement when practical feasibility factors are taken into account. Whenever available, corresponding published data are described. If not, the targets are based on database analysis of international development group members, on expert consensus and on feedback from external reviewers.

While the quality indicators in this document are most valid for patients receiving their complete treatment in the same center, where all technical requirements for treatment of cervical cancer are available, especially access to modern brachytherapy techniques, referral networks should be in place for centers that do not have access to brachytherapy techniques, with appropriate failsafe mechanisms to avoid lengthening of overall treatment time or waiting times between diagnosis and initiation of treatment. Centralization to these highly specialized centers is encouraged to ensure quality assurance and maximal effort at all levels.

Tumor stages are indicated following the International Federation of Gynecology and Obstetrics (FIGO) classification, updated in 2018, and the tumor node metastasis (TNM) classification, updated in 2021.^13^ Quality indicators 1–6 are general requirements related to pretreatment workup, time to treatment, upfront radiation therapy, and overall management, including active participation in clinical research and in the decision making process within a structured multidisciplinary team (Table 1). Quality indicators 7–17 are related to treatment indicators (Tables 2–3). Quality indicators 18 and 19 are related to patient outcomes (Table 4).

**Table 1:** General requirements

QI 1: Treatment decisions discussed at a multidisciplinary team meeting	
Type	Process indicator
Description	The decision for any therapeutic intervention (excluding diagnostic procedure, ie, biopsies or conization performed with a diagnostic intent) has been taken by a multidisciplinary team, including at least a gynecologic oncologist or specialized gynecologic surgeon dedicated to the management of gynecological cancer, a radiologist, a medical or clinical oncologist, a pathologist, and a radiation oncologist specialized in the treatment of gynecological cancers and with expertise in brachytherapy
Specifications	*Numerator*: number of cervical cancer patients for whom the decision for any therapeutic intervention has been reached within a multidisciplinary team *Denominator:* all patients with cervical cancer referred to that center
Target	≥95%
**QI 2: Required pretreatment workup**	
Type	Outcome indicator
Description	The minimal pre-radiotherapy workup for a histologically confirmed cervical cancer includes a clinical examination, pelvic MRI, and 18-FDG PET-CT
Specifications	*Numerator:* number of patients with histologically confirmed cervical cancer who receive a workup as defined above, before primary radiotherapy treatment (excluding palliative cases) *Denominator:* all patients with histologically confirmed cervical cancer treated with primary radiotherapy treatment (excluding palliative cases)
Target	≥90%.
**QI 3: Time to primary radiotherapy**	
Type	Outcome indicator
Description	Time between referral to the center and initiation of primary radiotherapy treatment
Specifications	*Numerator:* number of cervical cancer patients who start their primary radiotherapy treatment within 6 weeks from the date the patient is referred for the first time to the center *Denominator:* all patients with cervical cancer treated with primary radiotherapy treatment
Targets	Optimal target: ≥90% Minimum required target: ≥75%
**QI 4: Center participating in clinical trials in gynecological cancers**	
Type	Structural indicator
Description	The center participates in clinical trials in gynecological cancers involving radiotherapy
Specifications	*Numerator:* not applicable *Denominator:* not applicable
Target	At least one ongoing clinical trial or one clinical trial conducted in the past 5 years in gynecological cancers involving radiotherapy
**QI 5: Patients are treated with upfront radiotherapy and/or concurrent chemotherapy**	
Type	Outcome indicator
Description	Patients with node negative IB3–IIA2 not treated with surgery and patients with stage IIB–IVa cervical cancer are treated with upfront radiotherapy and/or concurrent chemotherapy
Specifications	*Numerator:* number of patients with the above criteria treated with upfront radiotherapy and/or concurrent chemotherapy (outside of a clinical trial) *Denominator:* total number of patients with the above criteria referred to a center (and treated outside of a clinical trial)
Target	≥95%
**QI 6: Patients are treated with brachytherapy boost**	
Type	Outcome indicator
Description	Patients treated with EBRT (with curative intent) for cervical cancer are treated with a brachytherapy boost
Specifications	*Numerator*: number of patients treated with EBRT (with curative intent) for cervical cancer treated with a brachytherapy boost *Denominator:* total number of patients treated with EBRT (with curative intent) for cervical cancer
Target	≥95%

EBRT, external beam radiotherapy; 18-FDG PET-CT, positron emission tomography–computed tomography with 2-deoxy-2-fluorine-18-fluoro-D-glucose; MRI, magnetic resonance imaging; QI, quality indicator.

**Table 2:** Treatment indicators

QI 7: Patients are treated with intensity modulated radiotherapy techniques	
Type	Outcome indicator
Description	Patients receiving pelvic and/or para-aortic radiotherapy are treated with IMRT-like techniques to decrease treatment related toxicity
Specifications	*Numerator*: number of cervical cancer patients treated with curative intent with pelvic and/or para-aortic IMRT per center
	*Denominator*: total number of cervical cancer patients treated with curative intent with pelvic and/or para-aortic external irradiation per center
Targets	Optimal target: 100%
	Minimum required target: ≥90%
**QI 8: Daily on-board IGRT and individualized margins are used to compensate for internal target motion**	
Type	Outcome indicator
Description	Patients are treated following an IGRT protocol with daily imaging based on on-board three-dimensional imaging (CBCT, MRI, or CT), with individual margins to compensate for internal target motion, daily verification modalities, and couch correction strategies. Replanning is performed when target motion has impact on dosimetric coverage
Specifications	*Numerator*: number of cervical cancer patients treated following an individualized IGRT protocol with daily on-board three-dimensional imaging
	*Denominator*: total number of cervical cancer patients receiving curative intent EBRT
Target	≥95%
**QI 9: Prescribed pelvic dose is 45 Gy in 1.8 Gy per fraction**	
Type	Outcome indicator
Description	Prescribed dose for pelvic and/or para-aortic EBRT is 45 Gy delivered in fractions of 1.8 Gy
Specifications	*Numerator*: number of patients treated with curative intent for cervical cancer and being prescribed a total dose of 45 Gy EBRT
	*Denominator*: total number of patients treated with curative intent EBRT for cervical cancer
Target	≥95%
**QI 10: Lymph node boosts are delivered in patients with macroscopic lymph node spread**	
Type	Outcome indicator
Description	Suspicious macroscopic lymph nodes are boosted, preferentially through SIB
Specifications	Lymph node boosts
	*Numerator*: number of patients with pelvic and/or para-aortic macroscopic lymph nodes treated with lymph node boost, excluding palliative cases
	*Denominator*: total number of patients with pelvic and/or para-aortic macroscopic lymph nodes treated with EBRT, excluding palliative cases
	SIB use
	*Numerator*: number of patients with pelvic and/or para-aortic macroscopic lymph nodes treated with SIB
	*Denominator*: total number of patients with pelvic and/or para-aortic macroscopic lymph nodes receiving lymph node boost
Targets	Lymph node boosts: ≥ 95%
	SIB use: ≥90%
**QI 11: Chemotherapy use**	
Type	Outcome indicator
Description	Patients with cervical tumor are treated with radiotherapy and concurrent chemotherapy
Specifications	*Numerator*: number of patients treated with curative intent EBRT for cervical cancer receiving concurrent chemotherapy
	*Denominator*: total number of patients treated with curative intent EBRT for cervical cancer who are fit for concurrent chemotherapy without contraindications, such as renal insufficiency, hematological comorbidities, etc
Target	≥95%
**QI 12: Imaging for IGABT**	
Type	Outcome indicator
Description	Patients are treated with IGABT and at least the first brachytherapy fraction is planned based on MRI with applicator in situ
Specifications	IGABT use
	*Numerator*: number of patients treated with uterovaginal brachytherapy having three-dimensional imaging (CT or MRI) with applicator in situ performed at each implant
	*Denominator*: total number of patients treated with uterovaginal brachytherapy
	MRI at least at the first fraction
	*Numerator*: number of patients treated with uterovaginal brachytherapy having an MRI with applicator in situ performed at least at the first fraction
	*Denominator*: total number of patients treated with uterovaginal brachytherapy without contraindications for MRI
Targets	IGABT use: 100%
	MRI at least at the first fraction: ≥60%
**QI 13: Combined intracavitary/interstitial brachytherapy use**	
Type	Outcome indicator
Description	Combination of intracavitary and interstitial implant technique is recommended in patients with advanced stages, poor response to chemoradiotherapy, and/or or large volume and/or asymmetric tumors. It also helps decreasing doses to organs at risk
Specifications	*Numerator*: number of patients treated with combination of intracavitary uterovaginal and interstitial brachytherapy
	*Denominator*: total number of patients treated with uterovaginal brachytherapy
Targets	Optimal target: ≥60%
	Minimum required target: ≥40%
**QI 14: Brachytherapy is delivered after the patient has received a total EBRT dose ≥36 Gy to allow maximal tumor regression**	
Type	Outcome indicator
Description	Brachytherapy is performed after the patient has received a total EBRT dose ≥36 Gy
Specifications	*Numerator*: number of patients having uterovaginal brachytherapy performed after a total EBRT dose ≥36 Gy
	*Denominator*: total number of patients treated with uterovaginal brachytherapy
Target	>95%
**QI 15: Overall treatment time does not exceed 50 days**	
Type	Outcome indicator
Description	Overall treatment time, calculated from the first EBRT fraction to the last brachytherapy fraction (for high dose rate treatment) or pulse (for pulsed dose rate treatments), is ≤50 days. Overall treatment time calculation includes the delivery of lymph nodes boosts
Specifications	*Numerator*: number of patients treated with radiotherapy (and/or concurrent chemotherapy) plus brachytherapy boost and having overall treatment time ≤50 days
	*Denominator*: total number of patients treated with radiotherapy (and/or concurrent chemotherapy) plus brachytherapy boost, excluding those with occasional severe medical complications (eg, neutropenia requiring treatment disruption or concurrent infection)
Target	≥90%
**QI 16: Minimum required criteria for brachytherapy treatment planning**	
Type	Process indicator
Description	The center follows a protocol including, at minimum, the criteria for brachytherapy provided in Table 3
Specifications	*Numerator*: not applicable
	*Denominator*: not applicable
Target	Brachytherapy treatment planning meets criteria detailed in the table above
**QI 17: Number of patients treated with EBRT plus brachytherapy per center per year**	
Type	Structural indicator
Description	A minimum number of patients treated per year per center with EBRT (and/or concurrent chemotherapy) plus brachytherapy
Specifications	*Numerator*: number of patients treated with EBRT (and/or concurrent chemotherapy) plus brachytherapy for cervical cancer per center per year
	*Denominator*: not applicable
Targets	Optimal target: n ≥20
	Minimum required target: n≥10

CBCT, cone beam computed tomography; CT, computed tomography; EBRT, external beam radiotherapy; IGABT, image guided adaptive brachytherapy; IGRT, image guided radiotherapy; IMRT, intensity modulated radiotherapy; MRI, magnetic resonance imaging; QI, quality indicator; SIB, simultaneous integrated boost.

**Table 3:** Brachytherapy dose volume histogram achievability

Target dose	D_90_ CTV_HR_	D_98_ CTV_HR_	D_98_GTV_res_	D_98_CTV_IR_		
	EQD2_10_	EQD2_10_	EQD2_10_	EQD2_10_		
Achieved in 70% of patients*	>90 Gy	>80 Gy	>95 Gy	> 60 Gy		
	<95 Gy					
Achieved in 90% of patients*	>85 Gy	>75 Gy	>90 Gy	–		
**OARs**	Rectum D2cm^3^ EQD2_3_	Bladder D2cm^3^ EQD2_3_	ICRU rectovaginal point EQD2_3_	ICRU bladder point EQD2_3_	Bowel D2cm^3^ EQD2_3_	Sigmoid D2cm^3^ EQD2_3_
Achieved in 70% of patients*	<65 Gy	<80 Gy	<65 Gy	<75 Gy	<65 Gy	<70 Gy
Achieved in 90% of patients*	<75 Gy	<85 Gy	<75 Gy	<85 Gy	<75 Gy	<75 Gy

*Achievability is assessed per dose volume histogram parameter.

CTV_HR_, high risk clinical target volume; CTV_IR_, intermediate risk clinical target volume; D_90_, minimal dose delivered to 90% of the target volume; D_98_, minimal dose delivered to 98% of the target volume; D_2cm3_, minimal doses delivered to the most irradiated 2 cm^3^ parts of the organs; EQD2, equivalent doses per fractions of 2 Gy with alpha/beta value of 3 Gy for late normal tissue reactions (EQD2_3_) and 10 Gy for tumor (EQD2_10_); GTV^res^, residual gross tumor volume at time of brachytherapy; ICRU, International Commission on Radiation Units and Measurements; OARs, organs at risk.

**Table 4:** Indicators related to patient outcomes

QI 18: A structured follow-up program of patients outcome is available	
Type	Outcome indicator
Description	All disease related events (including local failures) and grade ≥3 genitourinary and/or gastrointestinal and/or vaginal complications occurring after treatment are monitored in a structured program
Specifications	*Numerator*: not applicable
	*Denominator*: not applicable
Target	Availability of a structured follow-up program monitoring all disease related events and severe complications, as defined above
**QI 19: Patients are offered a sexual rehabilitation program**	
Type	Outcome indicator
Description	A structured holistic program for sexual rehabilitation relies on the identification of healthcare professionals specialized in the treatment of radiation induced side effects, including clinicians with expertise in sexual health, either in the center itself or through well identified referral networks
Specifications	*Numerator*: patients without local failure who are offered a sexual rehabilitation program
	*Denominator*: total number of patients without local failure
Target	≥80%

QI, quality indicator.

### General Requirements

The role of the multidisciplinary team in defining the best strategy before any treatment throughout the entire patient’s journey is pivotal to achieve high standards of care with multidisciplinary input and to decrease treatment disparities.^4–6^ One of the main objectives is to avoid the cumulative morbidity of surgery and radiotherapy. The multidisciplinary team meeting guides the optimum treatment for the individual patient, taking into account all available prognostic factors for tumor control (histology, tumor stage, patient's previous history, results from a comprehensive clinical and radiological staging), as well as the potential impact of treatments on functional outcome.

The appropriate pretreatment workup required before any treatment decision is detailed in the joint ESTRO–ESGO–ESP guidelines.^4–6^ A comprehensive tumor description is also crucial to guide radiotherapy volumes. A thorough pelvic examination should assess the local extension of the disease. Clinical drawings of tumor extent are useful tool as part of image guided radiotherapy protocols.^14^ In selected cases, examination under anesthesia may be necessary, and cystoscopy or proctoscopy should be considered if lesions in the urinary bladder or rectum are suspected on imaging. The locoregional extent of the disease should be assessed through magnetic resonance imaging (MRI). Before external beam radiotherapy, positron emission tomography–computed tomography with 2-deoxy-2-fluorine-18-fluoro-D-glucose (18-FDG PET-CT) is the optimal tool for assessment of nodal and distant disease in patients with extracervical tumor spread and to guide radiotherapy boosts. Surgical para-aortic lymph node staging is optional to guide radiotherapy volumes in patients with pelvic lymph node metastasis but negative para-aortic findings on 18-FDG PET-CT.^15 16^

Time for workup should be as short as possible to avoid delays in treatment initiation, even though data on the optimal cut-off are scarce and remain under investigation.^17–19^ Retrospective cohorts showed the prognostic impact of treatment initiation delays, although no definitive threshold exists.^19^ This time interval relates to various parameters, such as the time interval from diagnosis to first specialist assessment, time required for completing the pretreatment workup, and waiting lists before treatment. In an effort to minimize the time to treatment, it is necessary to anticipate and schedule radiotherapy as soon as possible, without waiting for completion of the previous stage to plan it (especially for patients who undergo a primary para-aortic lymph node dissection).

Clinical research is a major path to improve the quality of care and is a surrogate marker for the expertise, specialization, and dedication of a center. Patients treated in research hospitals conducting trials have a higher probability of receiving standard care compared with those treated in centers not participating in clinical studies. Possible background for that may be a more robust and adequate infrastructure and also participation in quality assurance programs.^20 21^ More specific to cervical cancer, it was shown in the International Study on MRI Guided Brachytherapy in Locally Advanced Cervical Cancer (EMBRACE) that adherence to protocols translated into improved outcomes through dissemination of modern brachytherapy concepts and implementation of image guided treatments.^22^ Participation in clinical trials is associated with quality assurance processes, including dummy runs and individual case review, and increases treatment quality following up-to-date standards.^22–25^ The benefit of such processes also extends to those patients not enrolled in study protocols.

The standard treatment for patients with node negative IB3–IIA2 cancers not treated with surgery and for patients with stage IIB–IVA cancer in the absence of distant metastatic disease is concurrent chemoradiotherapy plus brachytherapy.^26–28^ Omission of a brachytherapy boost from the treatment package is associated with poorer overall survival.^29 30^Brachytherapy is the only radiotherapy modality that allows dose escalation to >85–90 Gy without exceeding organs at risk dose constraints and should not be replaced by any external radiotherapy, including intensity modulated radiotherapy or stereotactic body radiotherapy techniques.^31 32^Several randomized studies have failed to demonstrate the benefit of neoadjuvant chemotherapy before external beam radiotherapy.^33^

### Treatment Indicators

There are well described dose–effect associations for treatment related toxicity due to irradiation for pelvic malignancies. Intensity modulated radiotherapy (including volumetric arc therapy and Helical Tomotherapy) has been shown to decrease radiation doses to the bowel and bladder, compared with historical three-dimensional conformal techniques, leading to better treatment tolerance (decrease in gastrointestinal and urinary toxicity) without impact on disease control.^33–35^ Intensity modulated radiotherapy is the preferred external irradiation technique in cervical cancer and the standard irradiation technique in international protocols.^4–6^ Some technical precautions are, however, necessary. Movements of the cervix and uterus due to bladder filling should be taken into consideration during radiation treatment planning, especially if complex contouring protocols based on multiple imaging series are used for definition of an internal target volume.^36 37^ Large variations in daily bladder filling require larger safety margins to consider the potential impact on the position of the clinical target volume. Various specifications do exist in terms of drinking protocols (timing and volume) as well as voiding. Minimizing the range of internal motion of the target volume using a protocol for bladder filling at the time of treatment planning and during all radiotherapy fractions gives the possibility to decrease the margins applied around the target volume. Decreasing interfaction movement may translate into a benefit in terms of radiation induced morbidity by reducing organs at risk doses. In addition, acquisition of multimodal imaging (MRI, 18-FDG PET-CT) applying the same protocol facilitates bony fusion for contouring protocols.

During fractionated radiotherapy, there may be major shifts in the clinical target volume, especially in the anterior–posterior and superior–inferior directions. These may have a significant dosimetric impact, especially with highly conformal techniques, such as intensity modulated radiotherapy.^38–40^ Ensuring adequate patient repositioning during a fractionated external beam radiotherapy course through image guided radiotherapy may improve the therapeutic index by allowing decreasing safety margins without comprising target coverage. The dosimetric advantages of image guided radiotherapy were demonstrated, although the clinical impact is still under investigation.

Advanced adaptive image guided radiotherapy currently relies on two strategies.^37 41–43^ The first consists of offline replanning strategies based on cone beam computed tomography monitoring; the second consists of an individualized library plan of the day integrated to the treatment workflow, with different specific internal target volume–T volumes applied according to the position of the target. Library plans are created using CT scans and/or MRI with variable bladder filling (and hence different uterine positions). For each day of radiotherapy treatment, an appropriate plan is chosen based on imaging on that day. Limitations of individual library plans should be taken into account. First, the correlation between bladder filling and uterus motion may change throughout the treatment. Second, the difficulty in preparing for variable rectal filling should be considered. Offline replanning strategies are more widely applied.^44^ These should not lead to treatment interruption. New technologies may furthermore allow for daily online replanning in the future.

Daily verification and couch correction is based on on-board three dimensional imaging (cone beam computed tomography, MRI, or CT) and registration on bony landmarks. In addition, daily monitoring of uterine and cervix movements is necessary to ensure that the clinical target volume is properly covered. This is a prerequisite before reducing the safety margins in the context of intensity modulated radiotherapy. Cone beam computed tomography is also necessary to consider re-contouring and replanning according to the motion patterns of the clinical target volume. Integration of MRI guided linear accelerator radiotherapy may lead to a better definition of the target on a daily basis without additional radiation exposure, potentially allowing refinement of internal target volume definition. A planning target volume margin is applied around the internal target volume to take into account set up errors.

There is no randomized study comparing 45 and 50 Gy external beam radiotherapy in cervical cancer. The total dose delivered to the high risk clinical target volume (CTV_HR_) and to organs at risk is the result of the sum of the dose delivered by external beam radiotherapy and by brachytherapy, with a significant impact of the relative contribution of each treatment. Numerous data suggest that the ability to achieve a dose distribution fulfilling all treatment planning objectives of the EMBRACE II protocol is improved when the external beam radiotherapy dose is 45 Gy compared with >46 Gy^45^.^46^ There is a direct association between the volume irradiated to 43 Gy during external beam radiotherapy and both acute and late bowel morbidity.^47^Finally, based on EMBRACE I data, it was not possible to identify any difference in nodal control between 45–46 Gy and >46 Gy schedules.^48^

In patients with 18-FDG PET-CT positive lymph nodes, correlations were shown between the total dose and the nodal control probability. Retrospective series reported a good oncological outcome and a low toxicity profile with simultaneous integrated boosts.^49–51^It was also demonstrated that larger lymph nodes require higher doses.^52 53^ Simultaneous integrated boosts should deliver a total (external beam radiotherapy plus brachytherapy) equivalent dose of around 60 Gy EQD2_10_ to suspicious macroscopic lymph nodes. The total lymph node doses should take into account the contribution of brachytherapy, which depends on applicator type, as well as implant geometry. Classically, the brachytherapy contribution is approximately 5 Gy for ilio-obturator lymph nodes, 2.5 Gy for common iliac lymph nodes, and not significant for para-aortic lymph nodes.^23 54 55^ The use of simultaneous integrated boosts to macroscopic lymph nodes is associated with significant reduction in high dose volumes compared with sequential boosting and high regional control and acceptable morbidity in the EMBRACE study, with 5 year nodal control of 87% (95% confidence interval 85% to 89%).^23 28^ Simultaneous integrated boosts avoid prolonging overall treatment time and therefore they are radiobiologically superior to the sequential approach (lymph node boosts delivered after brachytherapy). In addition, simultaneous integrated boosts are delivered concurrently with radiosensitizing chemotherapy. No direct comparison is available for simultaneous versus sequential boosts in cervical cancer patients. The sequential approach, however, increases overall treatment time and should be avoided when possible. Sequential boosts should be scheduled with minimal delay following brachytherapy. In the EMBRACE II study, lymph nodes considered as requiring external boost fulfilled the following criteria^56^:

1. Hypermetabolism suspected on 18-FDG PET-CT;
2. Short axis≥10 mm on scan or MRI;
3. Diameter between 5 and 10 mm on MRI with irregular contours, hypersignal, or rounded shape.

Randomized trials and large meta-analyses have demonstrated the benefit of concurrent chemoradiotherapy over radiotherapy alone, with a benefit in complete response rates (+10.2%), locoregional control (+8.4%), and overall survival (+7.5%).^57^ The benefit was seen among patients with stage I–II disease but also among patients with stage IIIb tumors.^57 58^ The number of chemotherapy cycles delivered along with external beam radiotherapy is a contributor to probability of cure. It appears from retrospective studies conducted in the era of image guided adaptive brachytherapy that administration of 5–6 full dose cycles of weekly cisplatin could reduce a patient’s risk of developing distant metastasis and/or local relapse.^58 59^ A benefit was reported with or without cisplatin use, but cisplatin 40 mg/m² weekly remains the most frequently proposed systemic treatment in combination with radiotherapy.^57^ In the case of contraindications or patient comorbidity, other chemotherapeutics and schedules may be considered (eg, weekly carboplatin area under the curve 2 if renal dysfunction). As the dose intensity of chemotherapy matters, it is crucial to have a clear protocol for dose or cycle modifications, and the number of chemotherapy cycles should be reported in the patient medical record.

A prospective multicenter study and numerous retrospective institutional series showed that brachytherapy based on three-dimensional imaging (CT or MRI) gives better local control rates, compared with X-ray based brachytherapy, while reducing late severe toxicities.^56 60 61^ GEC-ESTRO (Groupe Européen de Curiethérapie–European SocieTy for Radiation Oncology) published guidelines in 2005 to homogenize target volume definition, taking into account tumor extent at diagnosis and at the time of brachytherapy, mainly based on clinical examination and on MRI findings (T2 weighted sequence).^62^ Mature results of the prospective, observational multicenter cohort EMBRACE-I study were published with a median follow-up of 51 months (interquartile range 20–64). This study showed that application of image guided adaptive brachytherapy concepts was associated with a high local control probability of >90% across all stages with acceptable morbidity.^28^ Although MRI is the preferred imaging modality for image guided adaptive brachytherapy, its access is more limited than for CT which is frequently used to replace MRI for three-dimensional image acquisition with the applicator in place. However, CT has limited soft tissue contrast, leading to systematic overestimation of the tumor dimensions.^63^ Guidelines were published to adapt target volumes concepts to CT based three-dimensional imaging, but the gold standard still remains MRI guided image guided adaptive brachytherapy.^63^

One frequently used option is to perform MRI at a time point close to the brachytherapy procedure (without applicator), and to use it to adapt target volumes delineation, taking into account the overestimation of CT based delineation. The anatomical changes induced by the applicator may, however, lead to potential variations and deformations. Therefore, to minimize uncertainties, the first brachytherapy fraction should be planned based on the T2 weighted MRI sequence with the applicator in situ. According to MRI availability, MRI can be replaced by CT for succeeding fractions to verify the position of the application and its relationships with regard to organs at risk. In addition, each applicator insertion should be followed by acquisition of three-dimensional imaging and treatment replanning. While transabdominal and/or transrectal ultrasounds are useful to guide intrauterine and interstitial catheter placement, the possibility of replacing MRI with ultrasound for treatment planning in image guided adaptive brachytherapy procedures remains under investigation.^64^

For patients with cervical cancer treated with uterovaginal brachytherapy, the contribution of an intracavitary component is crucial to deliver high doses to the cervix. Performing an intracavitary implant implies placing an intrauterine tandem and a vaginal applicator, either commercial or based on a vaginal impression. Implantation of the brachytherapy applicator is carried out under aseptic conditions in an operating theater. Most often, the implantation is performed under general anesthesia or under spinal anesthesia, which provides appropriate pain management for patients, especially when the placement of paravaginal or parametrial interstitial catheters is necessary.^65–67^ In patients with limited tumor size at the time of brachytherapy, intracavitary brachytherapy is usually sufficient to achieve a good coverage of target volumes. However, in patients with unfavorable topography and/or with large residual tumors at the time of brachytherapy, the dose escalation process may be limited with intracavitary applicators alone. In order to achieve D_90_ CTV_HR_ >85–90 Gy in EQD2_10_ (equivalent doses per 2 Gy fractions, with alpha/beta value=10 Gy for tumor) without exceeding organs at risk dose constraints, combined intracavitary–interstitial brachytherapy use may be necessary in >40% of patients.^68–70^

Dose escalation is associated with a benefit in local control, especially among patients with advanced stages and/or poor response to chemoradiotherapy.^71^ Systematic usage of an interstitial brachytherapy component increases D_90_ CTV_HR_ from 83±14 Gy to 92±13 Gy (p<0.01), without increasing organs at risk doses. The 3 year local control rate in patients with a CTV_HR_ volume ≥30 cm^3^ was 10% higher (p=0.02) among patients treated with combined intracavitary–interstitial brachytherapy, compared with those treated with intracavitary brachytherapy only. Combined intracavitary–interstitial brachytherapy use does not increase the probability of late morbidity.^72^ Combined intracavitary–interstitial brachytherapy is required to deliver high doses and achieve high local control probability in patients with large residual tumors at the time of brachytherapy.^73^

The decision to perform only an intracavitary procedure or a combination intracavitary–interstitial application (and the choice of applicator) should rely on an individual pre-implant (possibly virtual) analysis taking into account dimensions, geometry, and topography of the CTV_HR_ as well as its relationships with organs at risk and patient individual anatomy. For combined intracavitary–interstitial techniques, a uterine tandem should be inserted inside the uterine cavity. The placement of interstitial catheters in addition to the uterine tandem is usually necessary to achieve proper dose distribution in the case of infiltrative tumors with persistent substantial residual tumor rest within the parametrium after external beam radiotherapy. The relative contribution of the intracavitary and interstitial components depends on each specific situation. Because complex interstitial brachytherapy procedures carry a risk of perioperative and/or postoperative complications, the healthcare facility should have the capacity for perioperative care and a gynecological emergency unit.

Tumor regression during external beam radiotherapy and/or concurrent chemotherapy contributes to minimize CTV_HR_volume, which is a major prognostic factor.^68 71 72 74–79^ This reduction is also an important factor to fulfill the dose coverage objective, since low volume CTV_HR_ volumes facilitate the dose escalation process, leading to higher D_90_CTV_HR_. Brachytherapy should be performed after 4–5 weeks of conventionally fractionated external radiotherapy to allow sufficient regression. At the same time, overall treatment time is a major parameter of therapeutic efficacy, and the total spread of the treatment should be limited as much as possible to avoid the phenomenon of accelerated repopulation. Several studies showed that the total treatment time, calculated from the first session of external beam radiotherapy until the end of brachytherapy, should be <50 days. Ideally, brachytherapy must therefore be scheduled no later than the seventh week.

Increased total treatment time is associated with an increased risk of local relapse.^72 80^If external beam radiotherapy and brachytherapy are carried out at different centers, the external beam radiotherapy center should ensure that the patient is referred sufficiently early in the treatment process (preferably before the start of external beam radiotherapy) so that the total overall treatment time is not prolonged. If such a process is not feasible, it is recommended that the patient is referred for her whole treatment in the center with the brachytherapy facility. Maximum tumor regression is achieved after the patient receives an external beam radiotherapy dose >36 Gy. Ideally, brachytherapy should be performed after the patient receives a minimum dose of ≥40 Gy but for logistic purposes, it is acceptable to schedule brachytherapy after the patient has received a total external beam radiotherapy dose >36 Gy. Such organization may help in keeping the overall treatment time as short as possible in patients with low volume or well responding tumors. For patients with advanced disease, the maximum external beam radiotherapy dose should be delivered (45 Gy) to take advantage of tumor regression at the time of brachytherapy.

As both external beam radiotherapy and brachytherapy contribute to the total dose delivered to the patient, recording target and organs at risk dose volume parameters is crucial to assess quality of dose distribution, which is a major contributor to the probability of cure without sequelae. Large data from the retro-EMBRACE and EMBRACE studies as well as numerous institutional series have demonstrated correlations between major clinical endpoints, such as local control and organ specific morbidities, and treatment dose/volume parameters, leading to identification of dose constraint parameters for treatment optimization, and especially for brachytherapy treatment planning.^47 70 80–85^

Target volume dose is associated with local control probability, especially for patients with advanced stages, infiltrative tumors, non-squamous histology, and poor response after chemoradiotherapy.^71 86–88^ Significant correlations were demonstrated between late morbidity probability and bladder, rectal, and bowel dose volume parameters.^89^ Alongside volumetric parameters, International Commission on Radiation Units and Measurements (ICRU) recto-vaginal and bladder reference point doses and posterior–inferior border of symphysis points are significant risk factors for urinary, rectal, and vaginal complications.^90 91^Recording treatment dose volume histograms for both external beam radiotherapy and brachytherapy is a major prerequisite to assess treatment quality. Implementation of modern brachytherapy concepts is progressive, with a learning curve yielding to increase target volume doses while reducing organs at risk doses, which is associated with an improvement in the therapeutic index. Recording dose volume histograms for target volumes and organs at risk may also be used to monitor this optimization process.

After completing external beam radiotherapy and brachytherapy courses, the treatment report stored in the patient medical record should include all relevant information on treatment modalities and techniques, tumor regression, and patient tolerance, according to the version of Common Terminology Criteria for Adverse Events (CTCAE). Total external beam radiotherapy and brachytherapy doses should be reported in terms of EQD2. Minimum dosimetric data to be included are detailed in the ICRU report 89.^92^ The ability to fulfill minimum required criteria for brachytherapy treatment planning is reflecting the expertise level of the center, as it reflects the appropriateness of the implant technique and the dosimetric process.^70^

Concurrent chemotherapy use, overall treatment time <50 days, and brachytherapy use have been demonstrated as major prognostic factors for patient survival and cure.^30 57 72 80^ There is a correlation between compliance with these quality indicators and patient volume of the radiotherapy facility.^93–95^ Patients treated in non-academic facilities show more frequent protocol deviations in terms of treatment completion and overall treatment time, compared with patients treated in academic facilities. Patients treated in facilities treating three or fewer patients per year receive less frequently concurrent chemotherapy than those treated in higher volume facilities. This encourages centralization to high volume centers to decrease treatment disparities and promote high quality treatments.^93^

The importance of individual brachytherapy expertise should be acknowledged, especially for complex catheterizations (eg, after previous cone resection or in patients with substantial residual disease), complex interstitial procedures (eg, paravaginal or parametrial interstitial implants), and for sophisticated high tech image guided treatment planning. A learning period exists, especially for modern brachytherapy treatments integrating dose escalation concepts. There is a correlation between increased experience, ability to fulfill planning aims, and clinical outcome. Fulfillment of the planning aim for dose prescription improves with experience, in parallel with the 5 year event free survival probability. The ability to fulfill planning aims for dose prescription can be significantly increased with growing experience, translating into a benefit for patients.^96^

### Indicators Related to Patient Outcomes

Indicators related to patient outcomes are of great importance and reflect the quality of treatment. To obtain valid data for auditing and accreditation purposes, we recommend that centers develop and follow a structured follow-up program, to report on oncologic outcomes, including local control rates and treatment related complications.

Local relapse (or progression) in patients treated with radiotherapy (and/or concurrent chemotherapy) and a brachytherapy boost is associated with a poor prognosis. Only a minority of patients with a local relapse will benefit from salvage treatment (surgery or more rarely re-irradiation).^97^ There is increasing evidence that improvement in local control correlates with an increase in overall survival.^98^ In the EMBRACE I study, the 5 year probability of local relapse was <10%.^28^ Even for the most advanced tumors (CTV_HR_of 70 mL), the probability of local control >85% was achieved. Local control is therefore a benchmark of the quality of the treatment (overall treatment time, use of concurrent chemotherapy, dose escalation, and interstitial brachytherapy use).

In parallel with local control, the rate of late complications is a robust marker of treatment quality, reflecting organs at risk dose exposure and the use of high quality brachytherapy implants (eg, interstitial applications to improve tumor coverage while minimizing the increase in irradiated volume and dose to organs at risk).^32^ Long term side effects should be documented in the medical record per organ site and scored according to the current CTCAE classification. It is important to assess the level of care by quality of life data to properly reflect treatment related morbidities. A high risk of underestimation of treatment related morbidity by clinicians was shown, and patient reported outcome measures should be integrated into the clinical routine.^99–102^

A structured program is necessary to report and review late gastrointestinal, urinary, and gynecological complications, including patient reported outcomes and quality of life, and to evaluate the true impact of treatments in terms of severe complications, but also mild to moderate morbidity. A structured global program for functional rehabilitation and holistic care should be available. Such programs rely on the identification of healthcare professionals specialized in the treatment of radiation induced side effects, including gynecologists, gastroenterologists, urologists, and psychological support, either in the healthcare structure itself or through well identified referal networks. Vaginal morbidity is the most frequent severe complication after pelvic irradiation for cervical cancer. Numerous patients report substantial radiation induced vaginal functioning problems. Mild to moderate vaginal stenosis and dryness may lead to sexual dysfunction and quality of life impairment. In addition, young patients treated with chemoradiotherapy also suffer from climacteric symptoms associated with definitive radiation induced menopause. Sexual health should be addressed, and any dysfunction should be documented in the medical record. Access to sexual rehabilitation programs should be available in the healthcare structure. Such rehabilitation programs involve medical and/or paramedical staff familiar with the prevention and palliation of long term radiation induced gynecological sequelae (eg, vaginal dilators, hormone replacement therapy, vaginal topicals, and psychological support).^103^

## Data Availability

All data relevant to the study are included in the article or uploaded as supplementary information.
