# Supplementary material for: ESGO/ESTRO quality indicators for radiation therapy of cervical cancer
Source: Int J Gynecol Cancer. 2023 May 29;33(6):862–75. doi: 10.1136/ijgc-2022-004180 (PMC10313976; doi:10.1136/ijgc-2022-004180)
Supplement: Supplementary data [file ijgc-2022-004180supp001.pdf]

SUPPLEMENTAL MATERIAL FILES

Supplemental appendix 1. List of the 99 external reviewers

Hoda Al-Booz, gynaecological oncologist (United Kingdom); **Anne-Sophie Bats**, gynaecological oncologist (France); **Virginia Benito**, gynaecological oncologist (Spain); **Margarida Bernardino**, gynaecological oncologist (Portugal); **Kjersti Bruheim**, oncologist (Norway); **Ewa Burchardt**, radiation oncologist (Poland); **Heloisa de Andrade Carvalho**, radiation oncologist (Brazil); **Supriya Chopra**, radiation oncologist (India), **David Cibula**, gynaecological oncologist (Czech Republic); **Abel Cordoba**, radiation oncologist (France); **Stefanie Corradini**, radiation oncologist (Germany); **Ovidiu Florin Coza**, radiation oncologist (Romania); **Astrid Agatha Catharina de Leeuw**, medical physicist (Netherlands); **Vitaliana De Sanctis**, radiation oncologist (Italy); **Catherine Dejean**, medical physicist (France); **Günter Emons**, gynaecological oncologist (Germany); **Beth Erickson**, radiation oncologist (United States of America); **Alexandre Escande**, radiation oncologist (France); **Sophie Espenel**, radiation oncologist (France); **Ana Felix**, pathologist (Portugal); **Lailatul Ferdous**, nurse (Bangladesh); **Daniela Fischerova**, gynaecological oncologist (Czech Republic); **Georgina Fröhlich**, medical physicist (Hungary); **Antonio Gil-Moreno**, gynaecological oncologist (Spain); **Deborah Gregory**, clinical oncologist (United Kingdom); **Lavanya Gurram**, radiation oncologist (India); **Cristina Gutierrez**, radiation oncologist (Spain); **Kathy Han**, radiation oncologist (Canada); **Matthew Harkenrider**, radiation oncologist (United States of America); **Kristina Hellman**, gynaecological oncologist (Sweden); **Gines Hernandez-Cortes**, obstetrician & gynaecologist (Spain); **Antonio Herreros**, medical physicist (Spain); **Peter Hoskin**, clinical oncologist (United Kingdom); **Nina Boje Kibsgaard Jensen**, clinical oncologist (Denmark); **Ina Jurgenliemk-Schulz**, radiation oncologist (Netherlands); **Ioannis Kalogiannidis**, gynaecological oncologist (Greece); **Kathrin Kirchheiner**, clinical psychologist, sexologist (Austria); **Christian Kirisits**, medical physicist (Austria); **Manon Kissel**, radiation oncologist (France); **Pawel Knapp**, gynaecological oncologist (Poland); **Susan Lalondrelle**, radiation oncologist (United Kingdom); **Valentina Lancellotta**, radiation oncologist (Italy); **Fabio Landoni**, gynaecological oncologist (Italy); **Laura Lane**, therapeutic radiographer (United Kingdom); **Jacob Christian Lindegaard**, clinical oncologist (Denmark); **Tiziano Maggino**, gynaecological oncologist (Italy); **Katarina Majercakova**, radiation oncologist (Spain); **Aljosa Mandic**, gynaecological oncologist (Serbia); **Simone Marnitz**, radiation oncologist (Germany); **Claudia Mateoiu**, pathologist (Sweden); **Patrice Mathevet**, gynaecological oncologist (Switzerland); **Mary McCormack**, clinical oncologist (United Kingdom); **Vicky McFarlane**, clinical oncologist (United Kingdom); **Lucas Mendez**, radiation oncologist (Canada); **Miloš Mlynček**, gynaecological oncologist (Slovakia); **Asif Muzamil**, clinical oncologist (United Kingdom); **Raj Naik**, gynaecological oncologist (United Kingdom); **Dina Najjari Jamal**, radiation oncologist (Spain); **Esten Nakken**, radiation oncologist (Norway); **Peter Niehoff**, radiation oncologist (Germany); **Felipe Ojeda**, gynaecological oncologist (Spain); **Brigida Pappalardi**, radiation oncologist (Italy); **Elisabetta Perrucci**, radiation oncologist (Italy); **Adeline Petit**, radiation oncologist (France); **Primoz Petric**, radiation oncologist (Switzerland); **Bradley Pieters**, radiation oncologist (Netherlands); **Thomas Samuel Ram**, radiation oncologist (India); **Francesco Raspagliesi**, gynaecological oncologist (Italy); **Isabelle Ray-Coquard**, medical oncologist (France); **Sophie Renard**, radiation oncologist (France); **Eva Cornelia Rijkmans**, radiation oncologist (Netherlands); **Alexandros Rodolakis**, gynaecological oncologist (Greece); **Angeles Rovirosa**, radiation oncologist (Spain); **Sergio Schettini**, obstetrician & gynaecologist (Italy); **Barbara Segedin**, radiation oncologist (Slovenia); **Monica Serban**, medical physicist (Canada); **Cristiana Sessa**, medical oncologist (Switzerland); **Paul Sevelda**, obstetrician & gynaecologist (Austria); **Frank-André Siebert**, medical physicist (Germany); **Tayup Simsek**, gynaecological oncologist (Turkey); **Piero Sismondi**, obstetrician & gynaecologist (Italy); **Alejandro Soderini**, gynaecological oncologist (Argentina); **Sofia Spampinato**, medical physicist (Denmark); **Hana Stankusova**, radiation oncologist (Czech Republic); **Artem Stepanyan**, gynaecological oncologist (Armenia); **Simona Stolnicu**, pathologist (Romania); **Jamema Swamidas**, radiation oncologist (India); **Luca Tagliaferri**, radiation oncologist (Italy); **Karl Tamussino**, gynaecological oncologist (Austria); **Margit Valgma**, radiation oncologist (Estonia); **Jacobus van der Velden**, gynaecological

Chargari C, *et al. Int J Gynecol Cancer* 2023; 33:1–14. doi: 10.1136/ijgc-2022-004180

oncologist (Netherlands); **Marion P. Rvan Gellekom**, medical physicist (Netherlands); **Ignace Vergote**, gynaecological oncologist (Belgium); **René Verheijen**, gynaecological oncologist (France); **Lisa Vicenzi**, radiation oncologist (Italy); **Elena Villafranca**, radiation oncologist (Spain); **Henrike Westerveld**, radiation oncologist (Netherlands); **Pauline Wimberger**, gynaecological oncologist (Germany); **Catheryn Yashar**, radiation oncologist (United States of America).
